# African swine fever virus pC147L inhibits retinoic acid-inducible gene I-like receptors pathway via targeting MAVS-TRAF6 complex

**DOI:** 10.1186/s13567-026-01716-y

**Published:** 2026-03-23

**Authors:** Jiaxuan Lv, Huan Chen, Jiaona Guo, Yurun Miao, Shengqiang Ge, Jianjun Dai, Zhiliang Wang, Yong-Sam Jung, Yingjuan Qian

**Affiliations:** 1 Sanya Institute of Nanjing Agricultural University, Laboratory of Emerging Animal Diseases and One Health, Nanjing Agricultural University, Nanjing, Jiangsu Province China; 2 MOE Joint International Research Laboratory of Animal Health and Food Safety, College of Veterinary Medicine, Nanjing Agricultural University, Nanjing, Jiangsu Province China; 3 China Animal Health and Epidemiology Center, Qingdao, Shandong Province China

**Keywords:** African swine fever virus, pC147L, innate immunity, RIG-I-like receptor, MAVS, TRAF6

## Abstract

African swine fever virus (ASFV) suppresses the host’s innate immunity by interfering with pattern recognition receptors (PRRs) signaling pathways; however, the molecular mechanisms by which it inhibits PRR signaling remain poorly characterized. This study aimed to identify the ASFV-encoded protein pC147L. The pC147L, a subunit of the viral RNA polymerase, acts as a potent inhibitor of the Retinoic Acid-inducible Gene I (RIG-I)-like receptors (RLRs) pathway, which mediates the detection of viral double-stranded RNA. We used quantitative reverse transcription–polymerase chain reaction (qRT-PCR) and western blot assays, and demonstrated that pC147L specifically blocked RLRs-mediated interferon-β (IFN-β) production without affecting other antiviral pathways, such as TLR3 or cGAS-STING pathways. Mechanistically, pC147L targeted mitochondrial antiviral signaling protein (MAVS), the central adaptor of RLR signaling pathway, to disrupt downstream signal transduction. Co-immunoprecipitation assays revealed that pC147L physically interacted with both MAVS and TRAF6, preventing the formation of the MAVS-TRAF6 complex and subsequent TBK1 recruitment required for MAVS signalosome assembly. Furthermore, the overexpression and knockdown experiments demonstrated that pC147L enhanced ASFV replication by suppressing the type I IFN response in porcine alveolar macrophage (PAM) cells. Collectively, our findings revealed a positive role of pC147L during ASFV infection by promoting viral replication through suppressing innate immune response, presenting it as a potential target for vaccine development.

**Supplementary Information:**

The online version contains supplementary material available at 10.1186/s13567-026-01716-y.

## Introduction

African swine fever virus (ASFV) is a nucleocytoplasmic large DNA virus (NCLDV) belonging to the family *Asfarviridae*. It infects domestic pigs and wild boars, causing African swine fever. This fever is a fatal hemorrhagic disease with a mortality rate approaching 100% [1]. The global prevalence of ASFV, combined with the lack of effective antiviral drugs or vaccines, poses a significant threat to the global pig industry [2]. ASFV is a cytoplasmic virus that mainly targets macrophages and monocytes, with morphogenesis occurring in a perinuclear region known as the viral factory. The ASFV genome consists of linear double-stranded (ds) DNA ranging from 170 to 190 kbp, depending on the virus strain. The genome encodes more than 150 proteins playing crucial roles in morphogenesis, genome replication, transcription, and evasion of the host immune response [1, 3, 4]. Understanding the virulence factors of pathogens and their interactions with the host immune system is essential for effective vaccine development and disease control strategies.

Similar to other NCLDVs such as the vaccinia virus (VACV), ASFV uses its own transcription system to generate transcripts, enabling subsequent viral protein synthesis. The RNA polymerase encoded by ASFV shares significant structural similarity with eukaryotic RNA polymerase Ⅱ (Pol Ⅱ) and comprises eight subunits: pNP1450L, pEP1242L, pH359L, pD205R, pC147L, pD339L, pC122R, and pCP80R [5–7]. Among these subunits, NP1450L and EP1242L serve as the core components with catalytic activity. In contrast, the remaining subunits are accountable for polymerase complex assembly and assist the core components in fulfilling their functions. pC147L is homologous to Pol Ⅱ RPB6. It makes conserved contacts with pNP1450L and is predicted to enhance the assembly and/or stability of RNA polymerase [6], suggesting its potential role in viral replication.

As the first line of defense against pathogen invasion, the host innate immune system is activated through various pattern recognition receptors (PRRs) that detect pathogen-associated molecular patterns (PAMPs) [8]. Viral nucleic acids are detected in the cytoplasm by specialized pattern recognition receptors (PRRs). dsDNA is primarily sensed by cyclic GMP-AMP synthase (cGAS), whereas dsRNA is recognized by retinoic acid-inducible gene I (RIG-I)-like receptors (RLRs), including RIG-I and melanoma differentiation-associated gene 5 (MDA5) [9]. Upon recognizing their ligands, RIG-I and MDA5 undergo several post-translational modifications (PTMs) crucial for their activation. These modifications include dephosphorylation, conformational changes, and polyubiquitination. Polyubiquitination, particularly K63-linked polyubiquitination, is crucial for activating RIG-I and MDA5, as it facilitates their oligomerization. Following oligomerization, RIG-I and MDA5 translocate to the mitochondria, where they interact with mitochondrial antiviral signaling protein (MAVS), their common adaptor protein [10]. MAVS is composed of an N-terminal tandem caspase activation and recruitment domain (CARD), a proline-rich region in the middle, and a C-terminal transmembrane (TM) domain The TM domain anchors it to the outer membrane of mitochondria [11]. Upon receiving the viral signal from RIG-I or MDA5, MAVS undergoes aggregation, a process facilitated by tripartite motif protein 31 (TRIM31)-mediated K63-linked ubiquitination. This aggregation results in prion-like aggregate formation, which is essential for exerting antiviral effects [12].

Aggregated MAVS serves as a scaffold to recruit of downstream signaling molecules, forming a complex called the MAVS signalosome. Upon activation, MAVS directly interacts with TNF receptor-associated factors (TRAFs), including TRAF2, TRAF3, TRAF5, and TRAF6. These interactions are mediated through specific TRAF interaction motifs (TIMs) located within its proline-rich region and TM domain [13]. These interactions are crucial for the downstream signaling cascade leading to an effective immune response. Among the TRAFs recruited by MAVS, TRAF6 plays an indispensable role in RLR-mediated signaling by activating nuclear factor kappa-B (NF-κB) and interferon regulatory factor 7 (IRF7) [14]. TRAF6 is a multifunctional adaptor protein characterized by its N-terminal zinc RING and zinc finger domains, which confer E3 ubiquitin ligase activity, and a C-terminal TRAF domain that facilitates interactions with various other proteins [15]. The TRAF6 structure enables it to function both as an E3 ligase and as an adaptor molecule within the RLR pathway. Upon activation, TRAF6 undergoes autoubiquitination. During this process, it attaches K63-linked ubiquitin chains to itself and other substrates, such as the NF-κB essential modulator (NEMO). This ubiquitination is essential for activating downstream kinases, including IκB kinase α/β (IKKα/β) and TANK-binding kinase 1 (TBK1). Moreover, TRAF6 forms a pre-formed complex with TBK1 in its resting state. Upon activation of the RLR pathway, this pre-formed TRAF6-TBK1 complex is immediately recruited to the MAVS signalosome. The spatial proximity facilitated by this recruitment allows for the phosphorylation and full activation of TBK1. Upon this activation, TBK1 phosphorylates IRF3, which then dimerizes and moves to the nucleus, IRF3 initiates the transcription of type I interferon (IFN) and IFN-stimulated genes (ISGs), which are crucial for the antiviral response [16, 17]. Overall, the MAVS-TRAF6 complex serves as a crucial molecular bridge, connecting the detection of viral dsRNA by RLRs to the activation of downstream signaling cascades.

ASFV is a cytoplasmic virus that generates both dsDNA and dsRNA during infection, triggering the activation of the cGAS/STING and RLR pathways in the cytoplasm [18, 19]. ASFV suppresses the cGAS/STING pathway through various mechanisms to facilitate viral replication. For instance, pEP364R and pC129R cooperatively cleave 2’,3’-cGAMP to inhibit STING activation [20]. Additionally, pMGF505-7R promotes the degradation of STING through the autophagy pathway [21], and pMGF505-11R facilitates STING degradation through the ubiquitin–proteasome, lysosomal, and autophagy pathways [22]. However, studies on ASFV regulating the RLR pathway are limited. Current studies primarily focus on RIG-I and MDA5 as the two upstream PRRs. Mechanistic studies reveal that pI267L specifically disrupts Riplet-mediated K63-linked ubiquitination of RIG-I, effectively suppressing its activation [19]. In parallel, pMGF360-4L targets MDA5 for autophagic degradation, thereby attenuating downstream signaling [23]. At present, our understanding of ASFV modulates the RLR pathway limited.

In summary, our study aimed to identify the ASFV-encoded protein pC147L. It demonstrated that pC147L functioned as a negative regulator of type I IFN signaling by specifically targeting the RLR-mediated signaling. pC147L directly bound to both MAVS and TRAF6, disrupting their interaction and consequently inhibiting IFN-β production, thus promoting ASFV replication. These findings revealed a novel mechanism underlying the evasion of host immune responses by ASFV and highlighted pC147L as a promising target for developing ASFV vaccines.

## Materials and methods

### Cell culture, virus and plasmids

Porcine Kidney-15 (PK-15) cells and human embryonic kidney 293 (293T) cells were cultured in Dulbecco’s modified Eagle’s medium (DMEM) supplemented with 8% fetal bovine serum(FBS) (PAN Biotech, Aidenbach, BY, GER) and 1% penicillin–streptomycin solution (Beyotime Biotechnology, Shanghai, SHH, China) at 37℃ in a 5% CO_2_ incubator. Porcine alveolar macrophage (PAM) cells were isolated from healthy specific pathogen-free (SPF) Bama miniature piglets using a protocol adapted from a previously established method [24]. The isolated cells were cultured in DMEM supplemented with 10% FBS (Hyclone, Logan, UT, USA) and 1% penicillin–streptomycin solution (Hyclone, Logan, UT, USA) at 37 ℃ in a 5% CO_2_ incubator.

The ASFV China/LN/2018/1 strain (GenBank accession number OP856591.1, abbreviated as ASFV-CN2018), used in this study, was preserved by the China Animal Health and Epidemiology Center (CAHEC) in Shandong province, China. The vesicular stomatitis virus (VSV) was stored in our laboratory (One Health Laboratory in Nanjing Agricultural University).

The C147L gene, amplified from the genome of ASFV-CN2018, was cloned into the pcDNA3-2xFlag to generate pcDNA3-2xFlag-C147L. Swine MDA5, RIG-I, MAVS, TRAF6, TBK1, and IKKε were amplified from the cDNA of PK-15 cells and subsequently cloned into the pCAGGS-HA, pCAGGS-Flag, or pcDNA3-Myc vectors. The pcDNA4-HA-IKKα, pcDNA4-HA-IKKβ, pcDNA4-HA-IRF3, pcDNA4-HA-IRF3-5D, pcDNA4-HA-p65, and pcDNA3-Myc-Ub plasmids were available in our laboratory. MAVS and TRAF6 truncation mutants were amplified using primers listed in Table 1. Each amplified fragment was then cloned into the pCAGGS-HA vector using a homologous recombination kit (Vazyme Biotech, Nanjing, JS, China) according to the manufacturer’s instructions. The plasmids used in the dual-calciferous reporter assay were also available in our laboratory, as described in a previous study [25].

**Table 1:** **Sequence of the primers used in amplification of MAVS and TRAF6 truncation mutants**

Primers	Sequence (5’ → 3’)
MAVS-CARD forward	CGGGGTACCATGACGTTTGCCGAGGAC
MAVS-CARD reverse	CCGCTCGAGTCATGAATTCCGCAGCAGGCTG
MAVS△CARD forward	CGGGGTACCATGAACAGCCCCCCAGCTCCAC
MAVS-TM forward	CGGGGTACCATGAAAGTGCCTACTGGCTTGCTG
MAVS-TM reverse	CCGCTCGAGTCACTGGGGCAGGCGC
TRAF6-Zn RING forward	CGGGGTACCATGAGTCTGCTACATTGTGAAAACAG
TRAF6-Zn RING reverse	CCGCTCGAGTCACTCAAGATGCCTCAGTTCCATCTTG
TRAF6-Fingers reverse	CCGCTCGAGTCAGTCTGAGCAACAGCCAGAGG
TRAF6-TRAF forward	CGGGGTACCATGCAGAATTTCCAGGAAACCATTCAGCAG
TRAF6-TRAF reverse	CCGCTCGAGCTATGTCCCCGAGTCTGTACTTCG

### Reverse transcription PCR (RT-PCR) and quantitative real-time PCR (qRT-PCR)

Total RNA was extracted from PK-15 and PAM cells using a Simple P Total RNA Extraction Kit (Bioer Technology, Hangzhou, ZJ, China) and subsequently reverse-transcribed into cDNA using a HiScript II Q RT Kit (Vazyme Biotech). SYBR Green-based quantitative reverse transcription–polymerase chain reaction (qRT-PCR) (Vazyme Biotech) was performed using a Life Technology instrument to measure mRNA levels of IFN-β and β-actin in PK-15 cells, and IFN-β, B646L, CP204L, and β-actin in PAM cells. Additionally, RT-PCR was performed to determine the mRNA levels of IFN-β, ISGs, IL-6, TNFα, and β-actin in PK-15 cells. The primers used in RT-PCR and qRT-PCR are listed in Table 2.

**Table 2:** **Sequence of the primers used in RT-PCR and qRT-PCR**

Primers	Sequence (5’ → 3’)
Swine IFN-β forward	GATACCAACAAAGGAGCAGC
Swine IFN-β reverse	GAGCATCTCGTGGATAATCAATAC
Swine ISG56 forward	CTTGGAGGAGATTGAGTTC
Swine ISG56 reverse	ATACAGCCAGGCATAGTTG
Swine ISG54 forward	GGCAGAGAATGAAATGTGTGGGA
Swine ISG54 reverse	AGAGGCAGGCGAGATAGGAGCAG
Swine TNFα forward	CCACCAACGTTTTCCTCACT
Swine TNFα reverse	CCCAGGTAGATGGGTTCGTA
Swine IL-6 forward	AAGGTGATGCCACCTCAGAC
Swine IL-6 reverse	TCTGCCAGTACCTCCTTGCT
Swine β-actin forward	TGAACCCCAAAGCCAACCGTGAG
Swine β-actin reverse	AGTGGTGCGGCCAGAGGCGTACAGG
ASFV C147L forward	CGCAAACATTGGTCATTATACCC
ASFV C147L reverse	CCCATTTCCCTGGGGTTC
ASFV B646L forward	GCATCCCAGGGGATAAAATG
ASFV B646L reverse	GAATAGGTTTGCTTTGGTGC
ASFV CP204L forward	AGGAGACGGAATCCTCAGCA
ASFV CP204L reverse	GGGCTCTTGCTCAAACAACG

### Antibodies and reagents

The primary antibodies used in this study were obtained from Cell Signaling (Beverly, MA, USA). They included rabbit anti-phospho-TBK1 (serine 172), rabbit anti-phospho-p65 (serine 536), rabbit anti-p65, mouse anti-IκBα and rabbit anti-phospho-IκBα (serine 32) antibodies. The mouse anti-actin, mouse anti-TBK1, rabbit anti-IRF3, mouse anti-Myc and rabbit anti-HA were purchased from Proteintech (Wuhan, HB, China). The rabbit anti-phospho-IRF3 (serine 396) was purchased from Affinity Biosciences (Changzhou, JS, China). The rabbit anti-TRAF6 was purchased from Santa Cruz (Shanghai, SHH, China). The mouse anti-Flag antibody was purchased from Sigma-Aldrich (St. Louis, MO, USA). HRP-conjugated goat anti-mouse IgG (H + L) or anti-rabbit IgG (H + L) were purchased from Millipore (Billerica, MA, USA). A polyclonal antibody against pC147L was generated in mice by immunization with purified pC147L protein.

PolydA:dT, high molecular weight (HMW) poly(I:C), and low molecular weight (LMW) poly(I:C) were purchased from InvivoGen (San Diego, CA, USA). The protease inhibitor cocktail was purchased from Thermo Fisher Scientific (Waltham, MA, USA).

### Dual-luciferase reporter assay

The dual-luciferase assay was performed in triplicate following the manufacturer’s instructions. 293T cells were seeded at a density of 1 × 10^5^ cells per well in 24-well plates overnight and then co-transfected with pGL3-Luc containing different promoter sequences (200 ng), mixed with Renilla luciferase vector pCMV-RL (2 ng), pcDNA3-2xFlag-C147L (200 ng), and indicated expression plasmids (200 ng) for 24 h using Lipofectamine 2000 reagent (Thermo Fisher Scientific, Waltham, MA, USA). The cells were harvested to determine luciferase activity using a dual-luciferase assay kit (Promega, Madison, WI, USA) and an MD SpectraMax iD5 instrument.

### Western blot analysis

Whole cell extracts were subjected to SDS-PAGE and subsequently transferred to the nitrocellulose (NC) filter membrane (Pall Corporation, New York, NY, USA). The membrane was blocked with 3% skimmed milk in PBST (PBS with 0.5% Tween-20) and incubated with primary antibodies at 4 °C overnight. Following washing with PBST, the membrane was incubated with HRP-conjugated secondary antibodies at 4 °C for 4 h, washed again with PBST, and treated with ECL reagent for imaging using a UVITEC Alliance Q9 Advanced Imager (Uvitec, Cambridge, Cambs, UK). Densitometric quantification of protein bands was performed using ImageJ software.

### Co-immunoprecipitation (Co-IP) assay

Cells were harvested with a lysis buffer supplemented with a phosphatase inhibitor cocktail and then incubated overnight at 4℃ with Protein A/G Magnetic Beads (Selleck, Houston, TX, USA) for immunoprecipitation along with anti-HA or anti-Flag antibodies. Rabbit or mouse IgG was used as a negative control. After incubation, the beads were washed with ice-cold lysis buffer and then mixed with 2 × SDS sample buffer (2% SDS, 10% glycerol, 60 mM Tris–HCl (pH 6.8), 5% β-ME and 0.01% bromophenol blue) followed by denaturation at 98 °C for 10 min. The mixture was centrifuged at 5000 rpm for 1 min, and the supernatant was analyzed by western blot analysis.

### GST pull-down assay

MAVS and TRAF6 tagged with GST, and pC147L tagged with His were expressed in Escherichia coli BL21(DE3). The GST fusion proteins were incubated with glutathione-coated beads (PointBio, Shanghai, SHH, China) at 4 °C for 2 h. Meanwhile, the His-tagged pC147L protein was purified using a nickel affinity chromatography column (Sangon Biotech, Shanghai, SHH, China). Subsequently, the purified pC147L protein was combined with the GST-fused proteins immobilized on the glutathione-coated beads in an E1A binding buffer (containing HEPES (50 mM), NaCl (50 mM), EDTA (5 mM), NP-40 (0.1%), and glycerol (10%), pH 7.6) and incubated at 4 °C for 4 h. After five washes with the E1A binding buffer, the samples were treated with 2 × SDS sample buffer.

### Semi-denaturing detergent agarose gel electrophoresis (SDD-AGE)

The 293 T cells, co-transfected with pCAGGS-HA-MAVS and pcDNA3-2xFlag-C147L for 24 h, were harvested with a buffer A (10 mM Tris–HCl, pH 7.5, 10 mM KCl, 1.5 mM MgCl_2_, 0.25 M D-mannitol and a protease inhibitor cocktail) in the Dounce homogenizer through repeated grinding. The samples were centrifuged at 700 *g* for 10 min at 4 °C and the supernatant was subsequently centrifuged at 10 000 *g* for 30 min at 4 °C to isolate the crude mitochondria (P5), which was the pellet obtained after centrifugation. P5 was dissolved in the 1 × sample buffer (0.5 × TBE, 10% glycerol, 2% SDS and 0.001% bromophenol blue) and then subjected to a 1.5% agarose gel following electrophoresis in the running buffer (1 × TBE supplemented with 0.1% SDS) for 40 min at a constant voltage of 100 V at 4 °C. After electrophoresis, the proteins were transferred to the NC filter membrane and then analyzed using western blot.

### RNA interference

The PAM cells were seeded in 12-well plates at a density of 3.6 × 10^5^ cells per well and transfected with siRNAs targeting C147L or scrambled siRNA using Lipofectamine RNAiMAX (Thermo Fisher Scientific, Waltham, MA, USA). After 24 h of transfection, the PAM cells were infected with ASFV-CN2018 (MOI = 1) and incubated for 36 h. Subsequently, the cells were harvested for qRT-PCR or western blot analysis. The siRNAs used in this study are listed in Table 3.

**Table 3:** **siRNAs used in this study**

siRNA	Sense (5’ → 3’)	Antisense (5’ → 3’)
siC147L-1	AACGAGCGCAUUACCUCCAACGUUU	AAACGUUGGAGGUAAUGCGCUCGUU
siC147L-2	UAGAGCGCAACAAUUGGCAAUUAAU	AUUAAUUGCCAAUUGUUGCGCUCUA
siNC	UUCUCCGAACGUGUCACGU (dT) (dT)	ACGUGACACGUUCGGAGAA (dT) (dT)

### Determination of intracellular ASFV genome copies

An absolute quantitative PCR (qPCR) method was employed to quantify the number of intracellular ASFV genome copies. Briefly, total DNA was extracted from infected PAM cells using a commercial DNA extraction kit (TIANLONG, Xi’an, SN, China) according to the manufacturer’s instructions. Standard curves were generated using serial dilutions of a plasmid encoding the viral gene B646L. qPCR was performed using B646L primers listed in Table 2 and a SYBR Green detection system. The number of viral genome copies in each sample was calculated by comparing the Ct values to the standard curve, enabling accurate absolute quantification of intracellular ASFV DNA.

### Statistical analysis

All experiments were performed at least three times unless otherwise indicated. Data were presented as means ± standard deviations (SDs). Statistical significance between groups was determined using the Student’s *t* test in GraphPad Prism 7.0 software (La Jolla, CA, USA). *P < 0.05, **P < 0.01, and ***P < 0.001.

## Results

### pC147L downregulates the IFN-β production by inhibiting the RLR pathway

**Figure 1:**
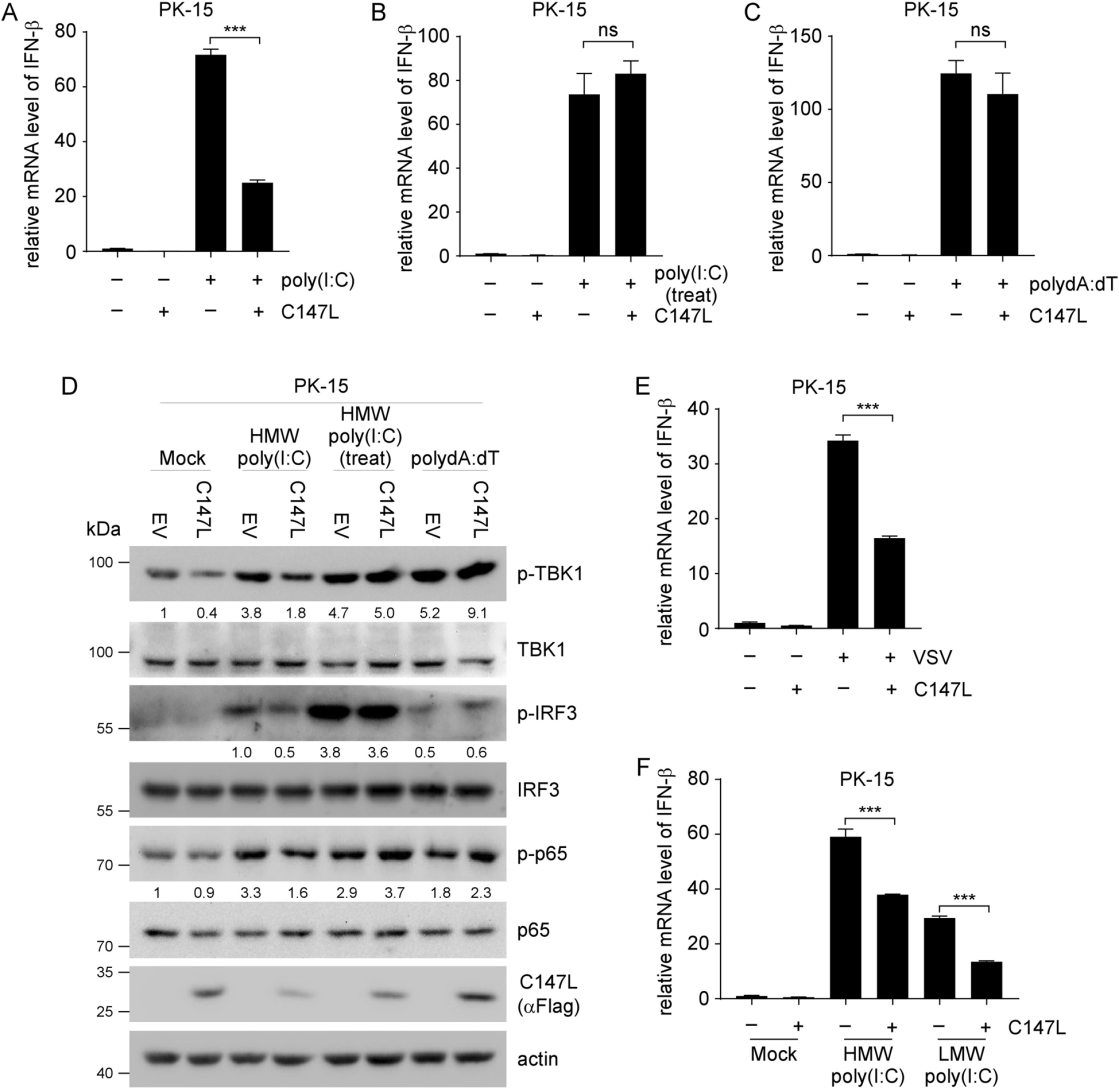
**pC147L downregulates IFN-β production by inhibiting the RIG-I like receptors pathway. A**–**C** PK-15 cells were transfected with either pcDNA3-2 × Flag-C147L (1 μg) or pcDNA3-2 × Flag (1 μg) for 24 h and subsequently stimulated as follows: **A** transfection with HMW poly(I:C) (2 μg/mL) for 2 h; **B** treatment with HMW poly(I:C) (10 μg/mL) for 2 h by direct addition to the culture medium; and **C** transfection with polydA:dT (2 μg/mL) for 6 h. Total RNA was isolated after stimulation, and IFN-β mRNA levels were quantified by qRT-PCR. **D** PK-15 cells were transfected and stimulated as described in (**A**–**C**), then lysed and analyzed by western blot with the indicated antibodies. **E** PK-15 cells were transfected with pcDNA3-2xFlag (1 μg) or pcDNA3-2xFlag-C147L (1 μg) were then infected with VSV (MOI = 1) for 8 h. IFN-β mRNA levels were quantified by qRT-PCR. **F** PK-15 cells transfected with either pcDNA3-2xFlag-C147L (1 μg) or pcDNA3-2xFlag (1 μg) for 24 h were subsequently transfected with HMW or LMW poly(I:C) (2 μg/mL) for 2 h. qRT-PCR was performed to assess the transcript levels of IFN-β.

Viruses have evolved multiple mechanisms to antagonize the host’s innate immune response to facilitate their replication. We conducted a dual-luciferase reporter assay by co-transfecting pGL3-Basic-IFN-β-Luc, pCMV-RL, pCAGGS-HA-MDA5, and expression plasmids encoding ASFV proteins in 293 T cells to identify potential virulence factors targeting the RLR pathway. The RLR pathway is a primary signaling pathway that recognizes cytosolic viral dsRNA. Among the 78 ASFV proteins screened, pC147L exhibited the strongest inhibition of IFN-β luciferase activity (Additional file 1), comparable to that of pMGF505-7R, a virulence factor that has been extensively characterized in previous studies [26, 27]. To further investigate the specific signaling pathway targeted by pC147L, we performed a qRT-PCR assay in which PK-15 cells were transfected with pC147L followed by various treatments with exogenous nucleic acid mimics. These treatments included transfection of poly(I:C) using Lipofectamine 2000 to activate the cytoplasmic RLR pathway, direct addition of poly(I:C) to the culture medium to stimulate the endosomal TLR3 pathway, and transfection with polydA:dT to engage the cGAS/STING pathway. The results demonstrated that pC147L selectively inhibited the IFN-β mRNA expression induced by the RLR pathway, while exhibiting no inhibitory effect on the TLR3 or cGAS/STING pathways (Figures 1A–C). This selective inhibition was further validated by using western blot assay, which revealed that pC147L effectively inhibited the phosphorylation of TBK1, IRF3, and p65 induced by transfection with poly(I:C) (Figure 1D). These findings were verified in the context of viral infection, using the vesicular stomatitis virus (VSV), which is a well-characterized agonist of the RLR pathway [28], to determine whether pC147L blocked the RLR pathway signaling. The PK-15 cells were transfected with pC147L for 24 h, and then infected with VSV (MOI = 0.1) for 8 h. Subsequent qRT-PCR analysis demonstrated that pC147L overexpression significantly suppressed VSV-induced expression of IFN-β (Figure 1E). Collectively, these results indicated that pC147L inhibited the RLR signaling pathway. Different molecular sizes of poly(I:C) were recognized by distinct sensors in the cytoplasm. The high molecular weight (HMW) poly(I:C) was primarily detected by MDA5, whereas the low molecular weight (LMW) poly(I:C) was predominantly sensed by RIG-I [29]. The regulation of the RLR pathway by pC147L was further elucidated by performing qRT-PCR and RT-PCR assay in which the PK-15 cells were first transfected with pC147L for 24 h and then with either HMW or LMW poly(I:C). The results revealed that pC147L suppressed the mRNA levels of IFN-β, ISGs and pro-inflammatory cytokines induced by both types of poly(I:C), notably, its inhibitory effect was more pronounced on LMW poly(I:C)-induced responses than on those triggered by HMW poly(I:C) (Figure 1F, Additional file 2). This suggested that pC147L effectively downregulated the IFN-β production by inhibiting the RLR pathway, probably targeting downstream components of MDA5 and RIG-I.

### pC147L inhibits IFN-β activation by targeting MAVS or upstream factors of the RLR pathway

**Figure 2:**
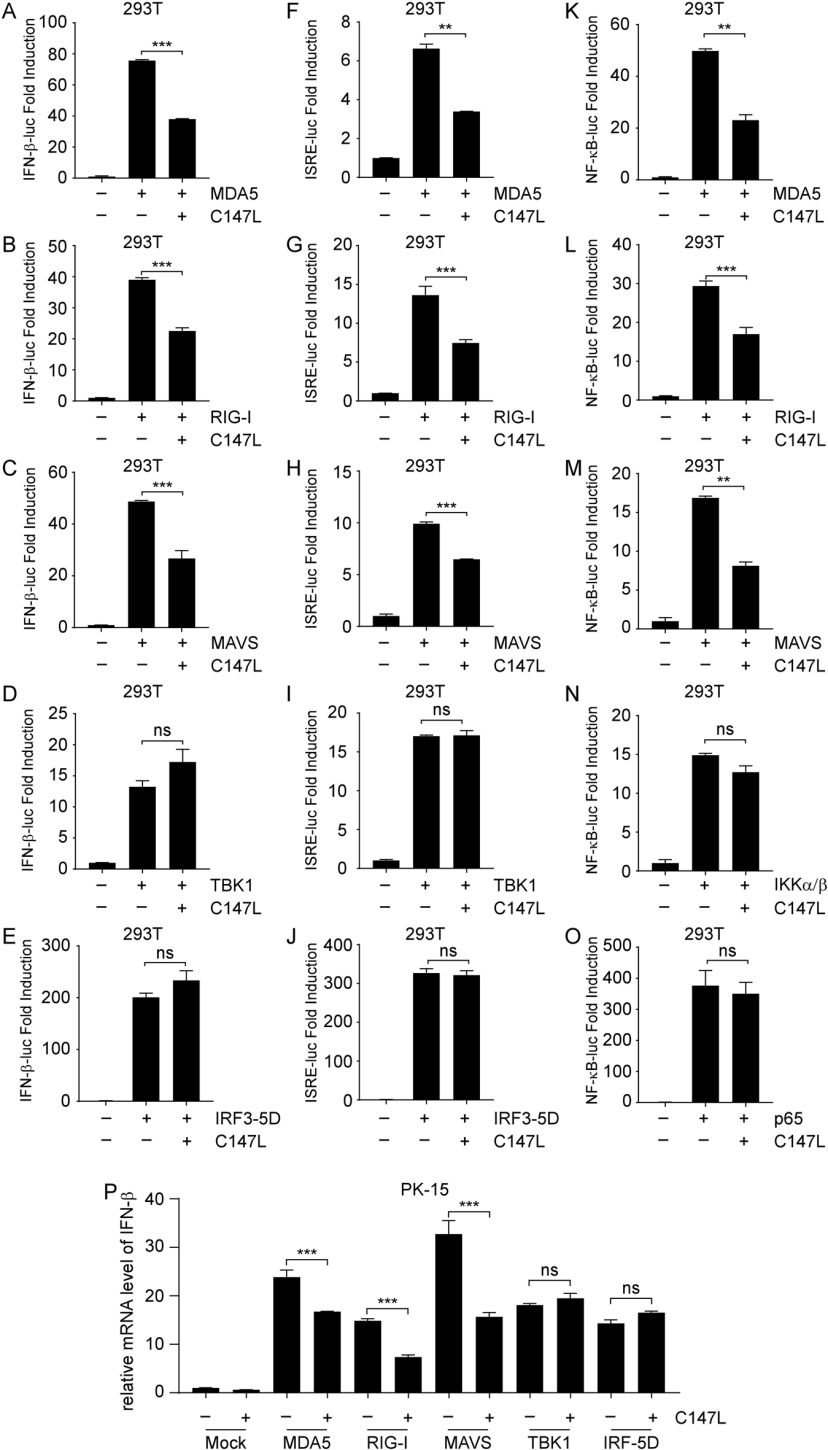
**pC147L inhibits IFN-β activation by targeting MAVS or upstream factors of the RLR pathway. A**–**L** 293 T cells were co-transfected with pCMV-RL (2 ng), pGL3-Basic-IFN-β-Luc (**A**–**E**), pGL3-Basic-ISRE-Luc (**F**–**J**), or pGL3-Basic-NF-κB-Luc (**K**–**O**) (200 ng), along with the indicated plasmids (200 ng) and pcDNA3-2 × Flag-C147L (400 ng). Luciferase activity was measured 24 h after transfection. **P** PK-15 cells were co-transfected with the indicated plasmids (400 ng) and either pcDNA3-2 × Flag or pcDNA3-2 × Flag-C147L (800 ng) for 24 h. Total RNA was extracted, and the mRNA levels of IFN-β was analyzed by qRT-PCR.

After sensing dsRNA ligand, MDA5 or RIG-I undergoes several PTMs, leading to the formation of oligomers. These oligomers then translocate to the outer membrane of mitochondria and interact with the adaptor protein MAVS, causing its aggregation. The aggregated MAVS subsequently recruits downstream molecules such as TRAFs, IKKs, TBK1, and IKKε, forming the MAVS signalosome. This complex mediates the phosphorylation and nuclear translocation of IRF3/7 and p65, leading to the transcription of IFN-β and pro-inflammatory cytokines [30]. We then identified the specific components targeted by pC147L using a series of luciferase assays by co-transfection of pC147L, expression plasmids encoding porcine RLR pathway molecules, and luciferase reporter plasmids. The results showed that pC147L inhibited the activity of IFN-β (Figures 2A–E), ISRE (Figures 2F–J), and NF-κB (Figures 2K–O) promoters induced by MDA5, RIG-I and MAVS, but not by downstream molecules of MAVS, including TBK1, IRF3-5D (constitutively active form of IRF3), IKKα/β and p65. These findings implied that pC147L likely targeted MAVS or upstream factors of the RLR pathway to inhibit signaling in the RLR pathway. This implication was further verified by assessing the impact of pC147L on the mRNA levels of IFN-β induced by the overexpression of various components of the RLR pathway. Consistent with the luciferase results, pC147L inhibited the IFN-β mRNA expression induced by the overexpression of MDA5, RIG-I and MAVS, but not by downstream TBK1 or IRF3-5D (Figure 2P). Taken together, these findings indicated that pC147L probably targeted MAVS or upstream factors of the RLR pathway to suppress RLR pathway signaling and prevent subsequent IFN-β production.

### pC147L attenuates MAVS-induced signaling without affecting MAVS activation

**Figure 3:**
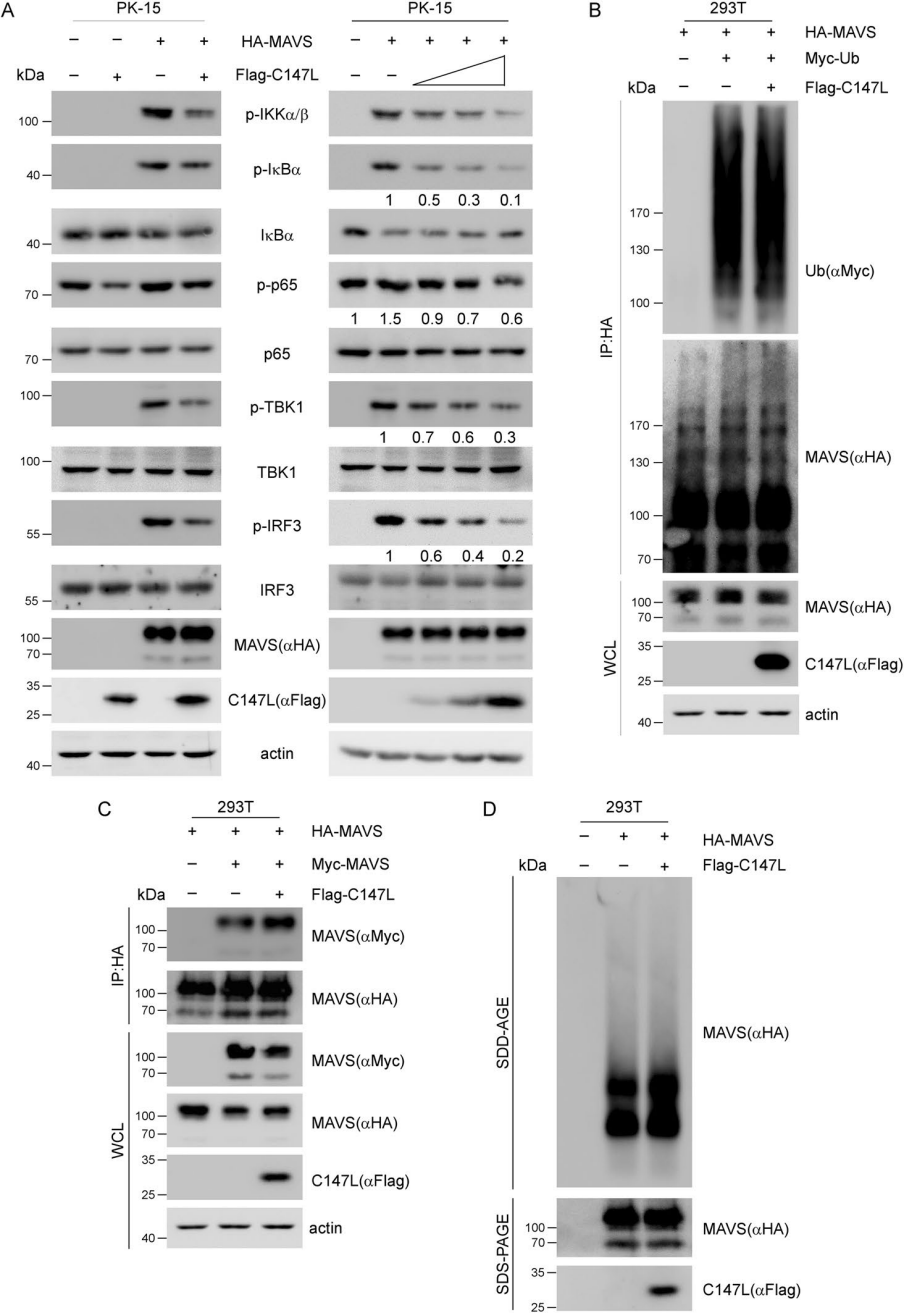
**pC147L attenuates MAVS-induced signaling without affecting MAVS activation. A** PK-15 cells were co-transfected with pCAGGS-HA-MAVS (300 ng) and pcDNA3-2 × Flag-C147L (300 ng, left panel; 300, 600, and 900 ng, right panel) for 30 h. Western blot analysis was performed using the indicated antibodies. **B** 293 T cells were co-transfected with pCAGGS-HA-MAVS (1 μg), pcDNA3-Myc-Ub (1 μg), and pcDNA3-2xFlag or pcDNA3-2xFlag-C147L (1 μg) for 30 h. The cell lysates were then subjected to immunoprecipitation with an HA antibody and analyzed by western blot using the indicated antibodies. **C** 293 T cells were co-transfected with pCAGGS-HA-MAVS (1 μg), pcDNA3-Myc-MAVS (1 μg), and either pcDNA3-2xFlag or pcDNA3-2xFlag-C147L (1 μg) for 30 h. The cell lysates were immunoprecipitated with an HA antibody and detected using western blot analysis with the indicated antibodies. **D** 293 T cells were co-transfected with pCAGGS-HA-MAVS (1 μg) along with pcDNA3 or pcDNA3-2xFlag-C147L (1 μg) for 30 h. The crude mitochondria (P5) isolated from the whole cell lysates were subsequently analyzed by SDD-AGE and SDS-PAGE with the indicated antibodies.

Upon receiving the signals transduced from MDA5 or RIG-I, MAVS was activated through PTMs, including phosphorylation and polyubiquitination, to form prion-like aggregates [11, 30]. We first performed a series of western blot assays with co-transfection of MAVS and pC147L in PK-15 cells to examine whether pC147L affected MAVS-induced signaling. The results indicated that pC147L significantly inhibited the phosphorylation of IKKα/β, IκBα, p65, TBK1 and IRF3 induced by MAVS overexpression (left panel in Figure 3A). Furthermore, the phosphorylation levels of these proteins decreased in a dose-dependent manner with increasing amounts of pC147L (right panel in Figure 3A). To further investigate whether pC147L affected MAVS activation and subsequently inhibited MAVS-induced signaling, we determined MAVS ubiquitination, as it is a prerequisite for MAVS aggregation. A co-immunoprecipitation (co-IP) assay following co-transfection of MAVS, Ubiquitin and pC147L in 293 T cells revealed that pC147L did not affect MAVS ubiquitination (Figure 3B). Based on this finding, we subsequently evaluated whether pC147L could directly interfere with MAVS aggregation. A co-IP assay was conducted following co-transfection of HA- and Myc-tagged MAVS and pC147L in 293 T cells. As shown in Figure 3C, pC147L exhibited no effect on the homologous interaction of MAVS. In accordance with this observation, the semi-denaturing detergent agarose gel electrophoresis (SDD-AGE) assay, which identifies components within crude mitochondrial extracts, confirmed that pC147L did not impair the formation of MAVS aggregates. Taken together, these findings suggested that pC147L attenuated MAVS-mediated signaling pathway without modulating its activation.

### pC147L interacts with MAVS and TRAF6

**Figure 4:**
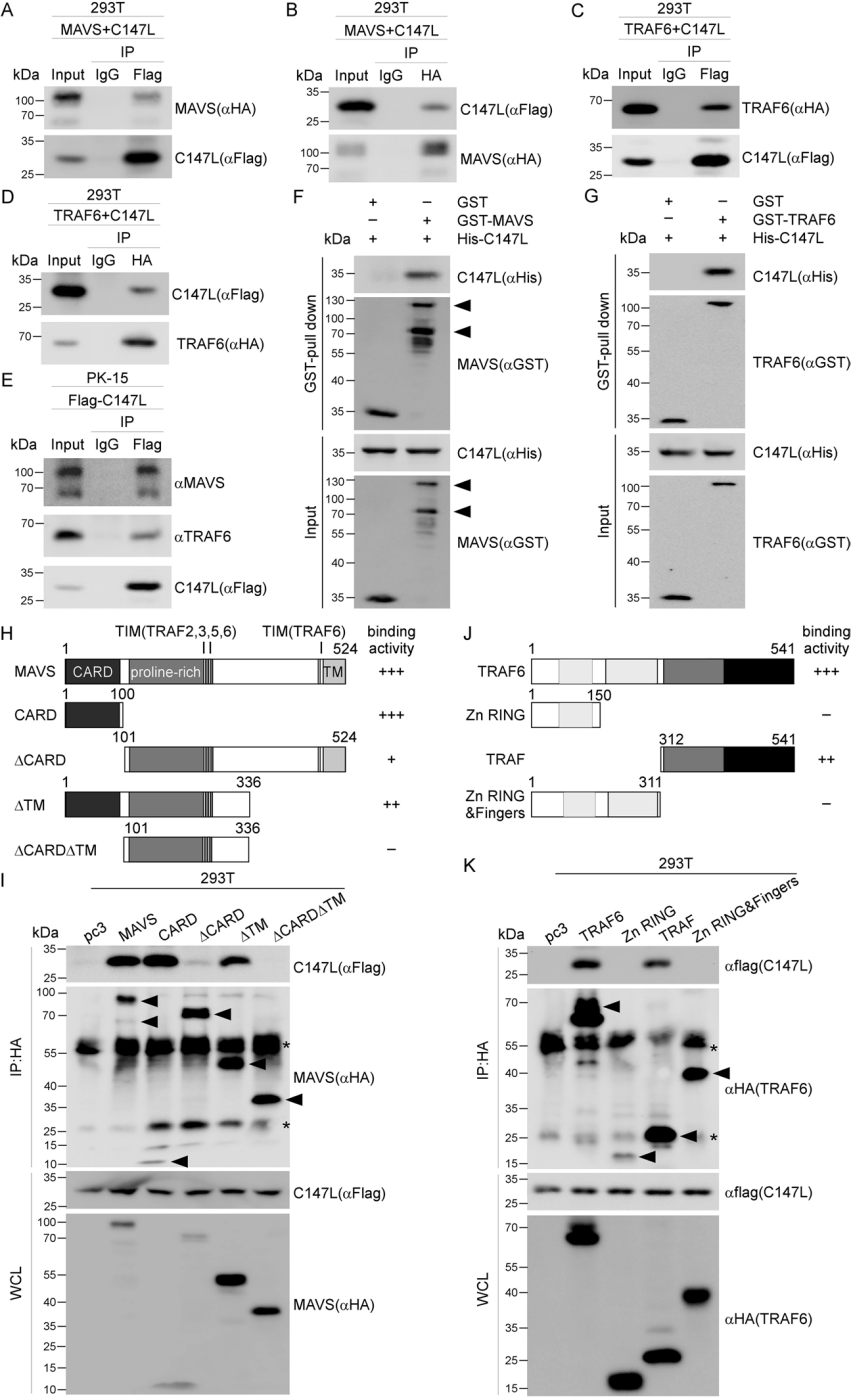
**pC147L interacts with MAVS and TRAF6. A**–**D** 293 T cells were co-transfected with pcDNA3-2 × Flag-C147L (1 μg) and pCAGGS-HA-MAVS (1 μg) (**A**, **B**) or pCAGGS-HA-TRAF6 (1 μg) (**C**, **D**) for 30 h. The cell lysates were subjected to immunoprecipitation with control IgG, Flag antibody (**A** and **C**), or HA antibody (**B** and **D**), followed by western blot analysis. **E** PK-15 cells were transfected with pcDNA3-2xFlag-C147L (1 μg) for 30 h, followed by transfection of HMW poly(I:C) (2 μg/mL) for 2 h. Immunoprecipitation was performed with Flag antibody and analyzed by western blot analysis. **F** and **G** Direct interactions between pC147L and MAVS or TRAF6 were examined using GST pull-down assays in *E. coli*. Briefly, GST-tagged MAVS or TRAF6 immobilized on glutathione-Sepharose beads were incubated with His-tagged pC147L at 4 ℃ for 4 h. Bound proteins were analyzed by western blot analysis with the indicated antibodies. **H**–**K** Truncated mutants of MAVS and TRAF6 are shown in **H** and **J**, respectively. The domain mapping of MAVS (**I**) and TRAF6 (**K**) was performed through an immunoprecipitation assay as described in **A**–**D**. Arrows indicate MAVS and TRAF6 constructs, and asterisks denote the heavy and light chains of the antibodies used for immunoprecipitation.

Activated MAVS directly interacted with several TRAFs through TIMs located within its proline-rich region and the C-terminal region. Specifically, the motifs PGENSE (153–158 aa) and PEENEY (455–460 aa) mediated binding to TRAF6, whereas PVQET (143-147aa) facilitated interactions with TRAF2, TRAF3, and TRAF5 [13, 31]. Among the TRAFs recruited by MAVS, TRAF6 played an independent role in the RLR signaling pathway by mediating the activation of NF-κB and IRF7 [14]. Given that pC147L did not impair MAVS activation, we proposed that pC147L likely disrupts the recruitment of TRAF6 to MAVS, thereby interfering with the downstream signaling cascade. Based on this speculation, we initially investigated the potential interaction between pC147L and MAVS or TRAF6 by conducting co-IP assays, wherein 293 T cells were co-transfected with HA-tagged MAVS or TRAF6 and Flag-tagged pC147L. The results showed that MAVS and TRAF6 were co-precipitated with pC147L using a Flag antibody (Figures 4A and C). Furthermore, pC147L was co-precipitated with MAVS and TRAF6 using an HA antibody (Figures 4B and D), indicating its interaction with both proteins. This interaction was further validated by an endogenous IP assay in PK-15 cells (Figure 4E). Given the known interaction between MAVS and TRAF6, we conducted an in vitro GST pull-down assay in *E. coli* to determine whether pC147L bound directly to MAVS or TRAF6. The results confirmed a direct interaction between pC147L and both MAVS and TRAF6, consistent with the co-IP findings (Figures 4F and G). Next, to identify the specific domains of MAVS and TRAF6 essential for interacting with pC147L, we generated truncated mutants of both proteins (Figures 4H and J). A series of co-IP assays were conducted by co-transfecting these truncations with pC147L in 293 T cells. Domain mapping analysis revealed that the CARD domain and the C-terminal region of MAVS, both of which contain TIMs crucial for TRAF6 binding, were indispensable for the interaction with pC147L (Figures 4H and I). Similarly, the C-terminal domain of TRAF6, which included a coiled-coil region (TRAF-N) and a β-sandwich motif (TRAF-C), was crucial for binding to pC147L (Figures 4J and K). Notably, the domains required for pC147L binding overlapped with those mediating the MAVS-TRAF6 interaction, suggesting that pC147L may competitively disrupt the formation of the MAVS-TRAF6 complex.

### pC147L disrupts the assembly of the MAVS-TRAF6 complex

**Figure 5:**
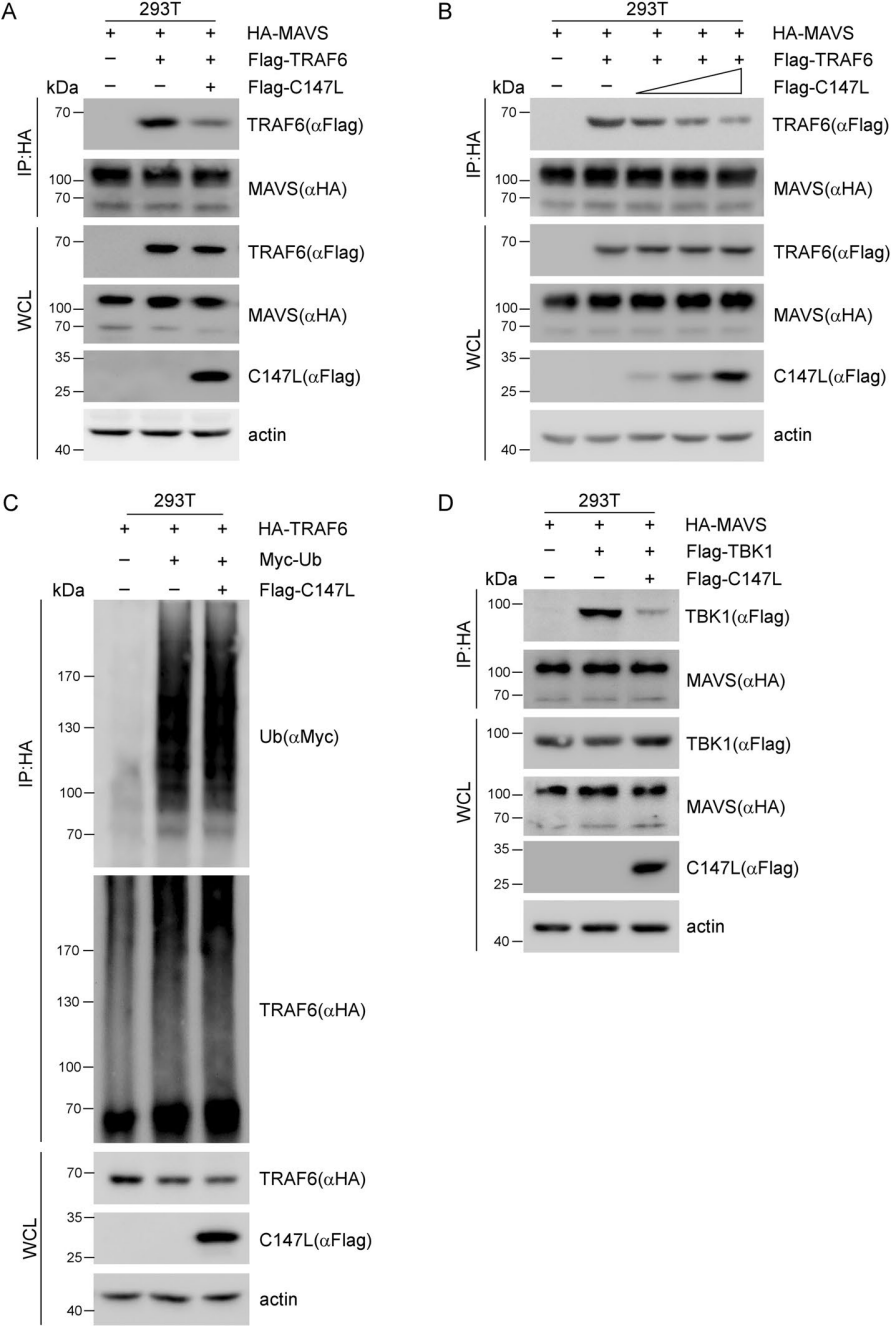
**pC147L disrupts the assembly of the MAVS-TRAF6 signalosome. A** 293 T cells were co-transfected with pCAGGS-HA-MAVS (1 μg), pCAGGS-Flag-TRAF6 (1 μg), and either pcDNA3-2 × Flag-C147L or pcDNA3-2 × Flag (1 μg) for 30 h. Immunoprecipitation was performed using an HA antibody and the samples were analyzed by western blot analysis. **B** The experiment was conducted similarly to panel (**A**) with an increasing transfection amount of pcDNA3-2xFlag-C147L (0.5, 1.0, and 1.5 μg). **C** 293 T cells were co-transfected with pCAGGS-HA-TRAF6 (1 μg), pcDNA3-Myc-Ub (1 μg), and either pcDNA3-2 × Flag or pcDNA3-2 × Flag-C147L (1 μg) for 30 h. The cell lysates were subjected to immunoprecipitation with an HA antibody and subsequently analyzed by western blot analysis using the indicated antibodies. **D** 293 T cells were co-transfected with pCAGGS-HA-MAVS (1 μg) and pCAGGS-Flag-TBK1 (1 μg), mixed with either pcDNA3-2xFlag-C147L or pcDNA3-2xFlag (1 μg) for 30 h. The cells were lysed, and immunoprecipitation was performed using an HA antibody. The immunocomplexes were then analyzed using western blot analysis.

Combining our previous findings that pC147L suppressed MAVS-mediated signaling pathway without affecting MAVS activation (Figure 3) with structural domain analysis, we hypothesized that pC147L disrupts the MAVS-TRAF6 interaction. We validate this by conducting co-IP assays in 293 T cells co-transfected with MAVS, TRAF6, and pC147L. The results showed that the interaction between MAVS and TRAF6 was significantly attenuated upon co-expression of pC147L (Figure 5A). Furthermore, we observed a dose-dependent suppression of MAVS-TRAF6 binding with increasing levels of pC147L expression (Figure 5B), suggesting that pC147L disrupts the assembly of the MAVS-TRAF6 complex. Besides, its role as an adaptor protein, TRAF6 functions as an E3 ligase in the signaling pathway. It uses its RING domain to first undergoes auto-ubiquitination, and then attaches K63-linked ubiquitin chains onto substrates, such as NEMO, thereby activating them [32]. To determine whether pC147L also modulates TRAF6 E3 ligase activity, we performed a ubiquitination assay. As shown in Figure 5C, pC147L did not affect TRAF6 auto-ubiquitination, which was consistent with our domain mapping data indicating that pC147L selectively binds to the TRAF domain but not to the Zn RING domain, which mediates its E3 ligase activity (Figure 4J). After directly binding to TRAFs, MAVS continued recruiting downstream molecules, including TBK1 and IKKε, and subsequently formed a signalosome to activate IRF3/7, ultimately inducing the transcription of type I IFN [16]. As pC147L attenuated MAVS-TRAF6 interaction, we speculated that pC147L probably also inhibits the recruitment of downstream molecules to MAVS. To verify this speculation, we performed a co-IP assay with co-transfection of MAVS, TBK1, and pC147L in 293 T cells. Our results indicated that pC147L impaired the recruitment of TBK1 to MAVS (Figure 5D). Collectively, pC147L disrupted the assembly of the MAVS-TRAF6 complex and blocked TBK1 recruitment to MAVS, thereby attenuating the formation of the MAVS signalosome.

### pC147L promotes ASFV replication

**Figure 6:**
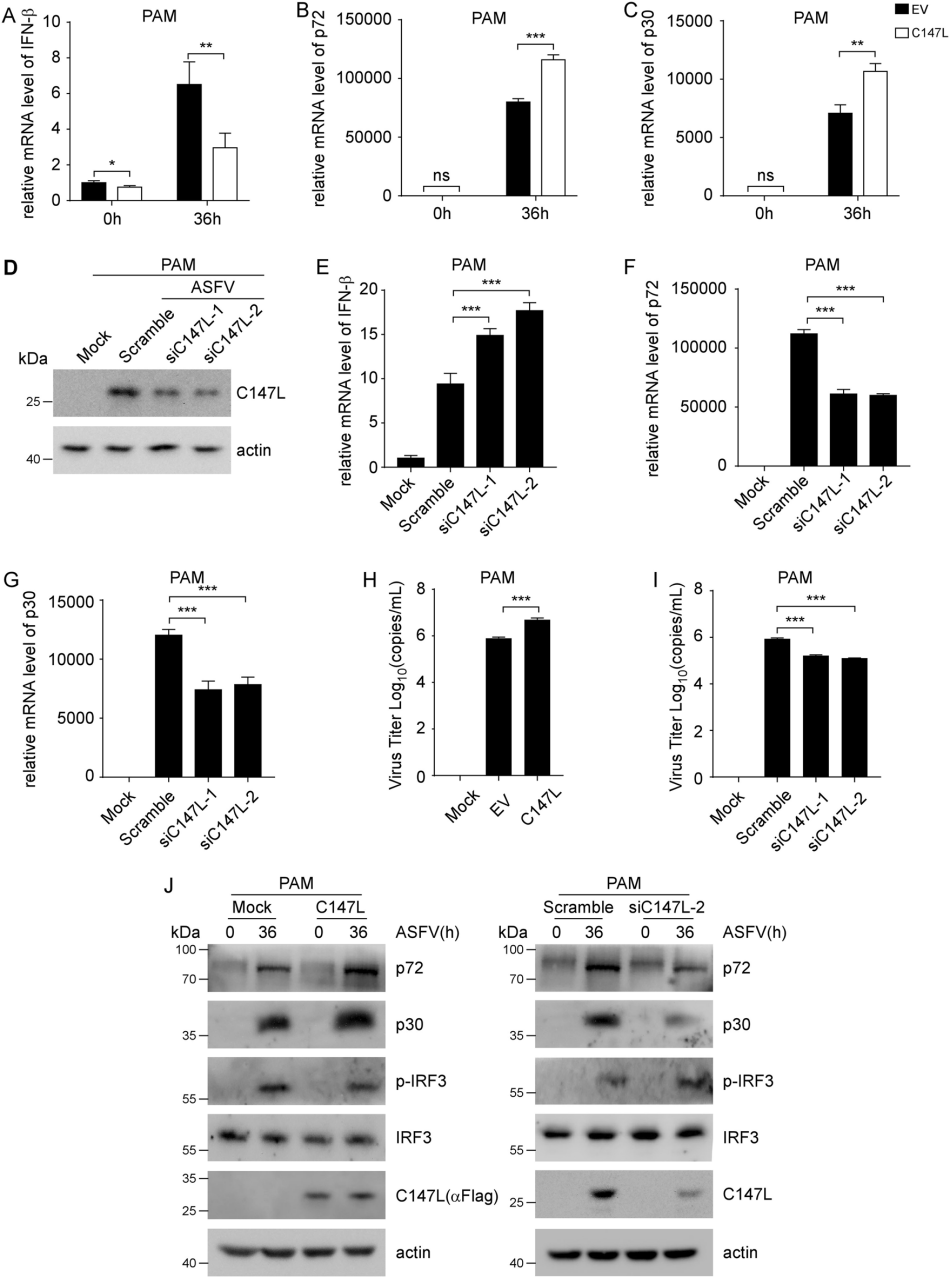
**pC147L promotes ASFV replication. A**–**C** PAM cells transfected with either pcDNA3-2xFlag (2 μg) or pcDNA3-2xFlag-C147L (2 μg) for 48 h were infected with ASFV (MOI = 1) for 36 h. The mRNA levels of IFN-β (**A**), B646L (**B**), and CP204L (**C**) were quantified by qRT-PCR. **D** PAM cells transfected with either scrambled siRNA or C147L-targeting siRNA (100 nM) for 24 h were infected with ASFV (MOI = 1) for 36 h. The protein levels of C147L were assessed by western blot analysis. **E**–**G** PAM cells treated as described in **D** were analyzed for IFN-β (**E**), B646L (**F**), and CP204L (**G**) mRNA levels through qRT-PCR. **H**–**I** Intracellular virus titer of PAM cells treated as described in **A** or **E** was determined. **J** PAM cells treated as described in panel **A** (left panel) or **E** (right panel) were harvested, and the whole cell lysates were analyzed using western blot analysis with the indicated antibodies.

Given that ASFV is a cytoplasmic virus and its protein pC147L suppresses RLRs pathway-dependent antiviral responses, we hypothesized that pC147L facilitates ASFV replication. To test this hypothesis, we first examined the impact of pC147L overexpression on ASFV replication in PAM cells. PAM cells transfected with pC147L were then infected with ASFV (MOI = 1) for 36 h. Subsequent qRT-PCR analysis revealed that pC147L overexpression significantly suppressed IFN-β transcription (Figure 6A), while enhancing the mRNA expression of viral genes B646L (encoding the major capsid protein p72) and CP204L (encoding the early structural protein p30) compared with vector controls (Figures 6B, C). Consistent with the qRT-PCR results, western blot analysis also showed that pC147L inhibited ASFV-induced IRF3 phosphorylation and upregulated p72/p30 protein expression (left panel, Figure 6J). To quantify viral replication, we performed a viral titer assay and found that pC147L increased the number of intracellular ASFV copies (Figure 6H). Taken together, these results suggested that pC147L facilitated ASFV replication by downregulating type I IFN responses. We investigated whether the knockdown of pC147L diminishes viral replication. Two siRNAs targeting C147L (siC147L-1 and siC147L-2) were designed. Their knockdown efficiency was confirmed by western blot analysis (Figure 6D). In siRNA-treated PAM cells, the knockdown of C147L significantly elevated IFN-β transcription (Figure 6E), while reducing the mRNA levels of B646L and CP204L (Figures 6F, G) compared with scrambled siRNA controls. This observation was further corroborated by western blot analysis (right panel, Figure 6J) and virus titer assays (Figure 6I), which confirmed the role of pC147L in promoting viral replication. Collectively, these findings demonstrate that pC147L promotes ASFV replication primarily by downregulating type I IFN responses, although its intrinsic role as a subunit of the viral RNA polymerase may also contribute to the observed reduction in viral replication upon knockdown.

## Discussion

PRRs are fundamental elements of the host innate immune system. They specifically recognize PAMPs and initiate a downstream signaling cascade that leads to the production of immune cytokines. RLRs, including RIG-I and MDA5, are crucial cytosolic sensors of viral dsRNA. Upon encountering dsRNA ligands, RIG-I and MDA5 undergo conformational changes that enable them to interact with their adaptor protein, MAVS [33]. Upon activation by either RIG-I or MDA5, MAVS oligomerizes on the outer mitochondrial membrane, creating a platform for the recruitment of TRAFs, TBK1 and IKKs. This leads to the activation of key transcription factors such as IRF3, IRF7, and NF-κB. Ultimately, this produces type I IFN and pro-inflammatory cytokines, establishing an antiviral state that limits viral replication and spread. Recent studies have reported that several ASFV proteins regulate the RLR signaling pathway to modulate the host antiviral responses. Specifically, pI267L and pE248R inhibit the K63-linked ubiquitination of RIG-I, thereby suppressing type I IFN production [19, 34]; pMGF360-4L promotes autophagic degradation of MDA5 to inhibit antiviral signaling [23]; pS183L suppresses type I IFN responses by preventing MDA5 oligomerization [35]; and pMGF505-9R enhances RLR signaling by promoting degradation of the host E3 ligase RNF125, which in turn stabilizes RIG-I, MDA5, and MAVS [36]. While multiple ASFV proteins have been shown to interfere with distinct components of the RLR pathway during ASFV infection, our study further expands this understanding by identifying pC147L as a novel viral antagonist of innate immune signaling. In our study, we used several exogenous nucleic acid mimics (poly(I:C) and polydA:dT) to trigger different innate immune signaling pathways. We observed that ASFV pC147L selectively downregulated IFN-β production through the RLR pathway, while leaving the TLR3 and cGAS/STING pathways unaffected (Figures 1A–D). Furthermore, the signaling cascade triggered by VSV, an agonist of the RLR signaling pathway, was also suppressed by pC147L overexpression (Figure 1E). We identified MAVS as the specific target of pC147L through luciferase reporter and RT-PCR assays (Figure 2).

MAVS serves as a central hub in the RLR signaling pathway, and several PTMs regulate its activation. Ubiquitination is one of the most significant PTMs that modulate MAVS activity. K63-linked ubiquitination of MAVS, primarily mediated by the E3 ligase TRIM31, facilitates MAVS oligomerization and the formation of prion-like aggregates [12]. Many viruses have evolved strategies to target MAVS and disrupt this critical signaling axis, thereby evading the host immune response. For example, Hepatitis C virus (HCV) encodes the NS3/4A protease, which cleaves MAVS at its mitochondrial localization site, effectively abolishing MAVS-dependent signaling and preventing the induction of type I IFNs [37]. Similarly, the non-structural protein NS4B of the Zika virus (ZIKV) has been shown to interact with MAVS, impairing its ability to form functional signaling complexes and inhibiting downstream type I IFN production [38]. Furthermore, the porcine reproductive and respiratory syndrome virus (PRRSV) targets MAVS for degradation through its nsp4 protein, thereby suppressing he RIG-I-mediated antiviral response [39]. Based on the PTMs of MAVS, we performed the ubiquitination and SDD-AGE assays. However, we found no effect of pC147L on MAVS ubiquitination or oligomerization (Figures 3B–D), suggesting that pC147L does not influence on MAVS activation.

MAVS contains several TIMs, specifically TRAF2, TRAF3, TRAF5, and TRAF6, which enable direct binding. These TRAFs function as E3 ubiquitin ligases, playing pivotal role in signal transduction by ubiquitinating various signaling proteins, thereby modulating their activity. The interaction between MAVS and TRAFs is crucial for the downstream activation of transcription factors such as IRF3, IRF7, and NF-κB [39, 40]. We used co-IP and GST pull-down assays and discovered that pC147L directly bound to both MAVS and TRAF6, specifically interacting with the CARD and C-terminal domains of MAVS, as well as the TRAF domain of TRAF6. Further co-IP assays revealed that pC147L disrupted the formation of the MAVS-TRAF6 complex (Figures 5A, B). Moreover, our preliminary results (data not shown) indicated that pC147L had no influence on TRAF3-mediated signaling, but might modulate the activation of TRAF2 and TRAF5 within the RLR pathway. Further studies are warranted to elucidate the molecular basis of these interactions. Interestingly, pC147L did not affect TRAF6 auto-ubiquitination (Figure 5C). Besides its role in the RLRs pathway, TRAF6 is also involved in other innate immune pathways, such as the cGAS/STING and TLR3, by exerting its E3 ligase activity to facilitate signal transduction. For example, TRAF6 promotes cGAS polyubiquitination, leading to its activation. Additionally, TRAF6 mediates K63-linked ubiquitination of glycogen synthase kinase 3β (GSK3β). This is a critical step for the formation of the TIR-domain-containing adaptor protein inducing IFN-β (TRIF)-assembled signaling complex, which amplifies the TLR3 signaling cascade [41, 42]. Our data suggested no influence of pC147L on IFN-β production induced by the cGAS/STING or TLR3 pathways (Figure 1). Taken together, these results indicated that pC147L specifically targeted TRAF6 by modulating its protein–protein interactions without affecting its E3 ligase activity.

Beyond its role in type I IFN production, MAVS plays a multifaceted role in key physiological processes, including apoptosis, autophagy, and the inflammatory response. Apoptosis and autophagy are two distinct but interconnected forms of programmed cell death essential for maintaining cellular homeostasis and responding to various stress signals. Upon activation by dsRNA recognition through RIG-I or MDA5, MAVS can induce apoptosis through a caspase-dependent pathway. Specifically, MAVS recruits and activates caspase-8. This process is facilitated by the activating molecule in Beclin-1-regulated autophagy protein 1 (AMBRA1). This activation subsequently leads to the cleavage and activation of caspase-3, culminating in apoptosis. The caspase activation and apoptotic function of MAVS require its CARD and TM domains, underscoring their essential roles in MAVS-mediated apoptosis [43–45]. The mechanism by which MAVS participates in autophagy is as follows. Upon recognition of dsRNA by RIG-I, the RIG-I-MAVS-TRAF6 signaling axis is activated. This activation leads to the TRAF6-induced K63-linked ubiquitination of Beclin-1, a critical regulator of autophagy. This process ultimately results in the induction of autophagy [46]. Furthermore, MAVS plays a positive role in inducing of a vital element of the inflammatory response. Upon viral infection or mitochondrial stress, MAVS can interact with NLRP3, promoting its oligomerization and activation. This interaction also leads to the recruitment and activation of caspase-1, which is responsible for the cleavage and maturation of pro-inflammatory cytokines such as intereleukin-1 β (IL-1β) and IL-18 [47, 48]. Through these mechanisms, MAVS regulates both innate immune responses and cellular death pathways during viral infection. ASFV employs diverse strategies to modulate apoptosis, autophagy, and inflammation in host cells. ASFV can both inhibit and induce apoptosis, depending on the infection stage and the specific needs of the virus. In the early stage, ASFV suppresses apoptosis to ensure that the infected cells remain viable long enough for the virus to replicate [49, 50]. As the infection progresses, ASFV may induce apoptosis in infected cells to facilitate the release of viral particles, promoting the spread of the infection [51]. Similar to its modulation of apoptosis, ASFV has been reported to both positively and negatively regulate autophagy by encoding various viral proteins [52–54]. Moreover, ASFV dampens the host inflammatory response by downregulating NF-κB activity and preventing inflammasome formation [11, 26, 55, 56]. The previous mentioned regulation of host physiological processes by ASFV enables the virus to persist within the host. pC147L may participate in the regulation of more potential biological processes by interacting with MAVS. Further experiments are needed to verify this hypothesis.

Many NCLDVs encode viral RNA polymerases (vRNAPs) that exhibit structural and functional similarities with their eukaryotic counterparts. The vRNAP encoded by ASFV shows a high degree of similarity to eukaryotic RNA polymerase II (Pol II) [57]. pC147L, which is homologous to the RPB6 subunit of Pol II, plays essential role in the assembly of vRNAP and in maintaining its conformational stability [6, 58]. However, the role of pC147L in the context of viral infection has not been reported. Our study demonstrated that overexpressing or knocking down pC147L during ASFV infection facilitated viral replication by inhibiting IFN-β production (Figure 6). Nevertheless, the impact of deleting pC147L on viral growth, as well as the structure and function of the vRNAP, still requires further verification through knockout experiments.

**Figure 7:**
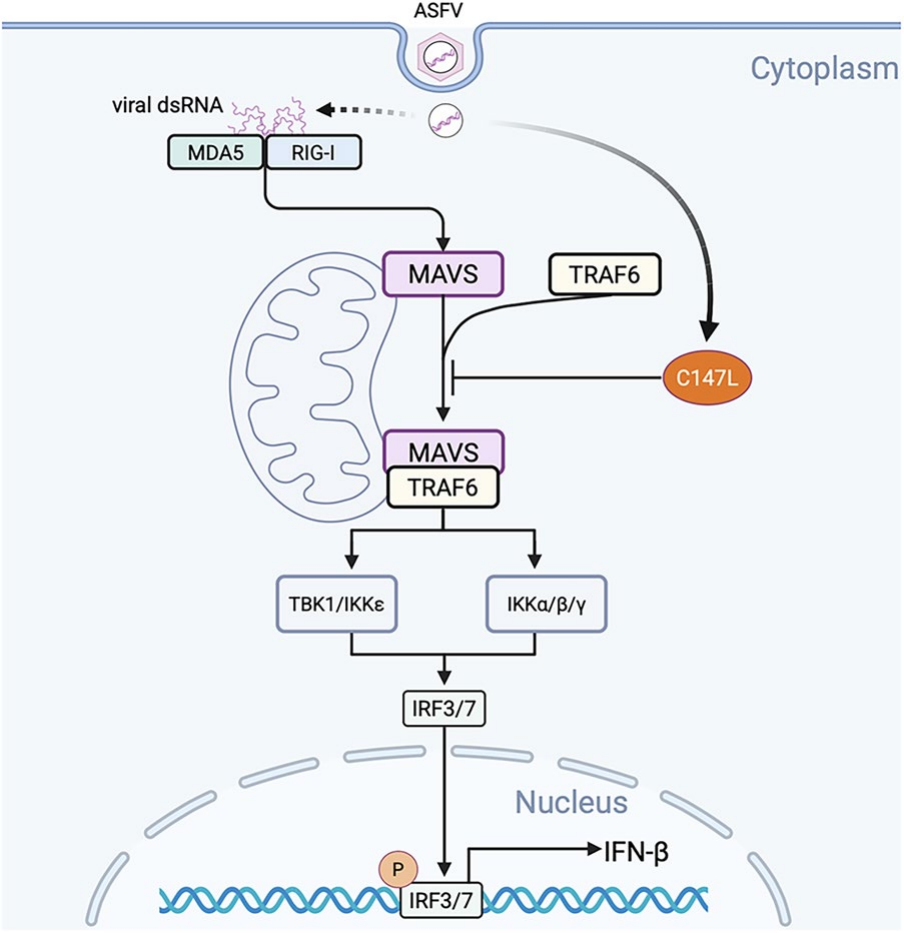
**Schematic model of the immune evasion mechanism mediated by ASFV pC147L.** ASFV pC147L disrupted the assembly of the MAVS-TRAF6 complex, thereby inhibiting the production of IFN-β induced by the RLR pathway.

To date, significant progress has been made in ASFV vaccines using live attenuated vaccines (LAVs). These vaccines involve the deletion of specific genes from ASFV to diminish its virulence while preserving its capacity to elicit immune responses. Genes such as I177L, 9GL, CD2v, MGF505, and MGF360 have been targeted for deletion, resulting in attenuated strains with promising protective efficacy [59–63]. However, LAVs carry the risk of reverting to their virulent form, potentially causing outbreaks if the vaccine strain regains pathogenic characteristics. Additionally, the long-term safety and stability of these vaccines are still under investigation. Single-cycle vaccines (SCVs) represent an innovative approach to vaccine development. They are designed to offer the immunogenic benefits of LAVs while minimizing the safety risks through the conditional expression of essential genes required for viral replication. VACV, another member of the NCLDVs, has been successfully engineered into SCVs by regulating the expression of structural proteins using tet operon elements [64]. Our study revealed that pC147L, the RPB6 subunit of vRNAP, as a positive regulator of ASFV infection by downregulating type I IFN response via disrupting the MAVS-TRAF6 complex assembly (Figure 7). Given its role in both immune evasion and viral replication, the loss of pC147L is expected to significantly impair ASFV replication efficiency. However, this impairment does not necessarily equate to a complete loss of replicative capacity under all conditions. Therefore, pC147L may not be suitable as a standalone deletion target, but its modification could still be incorporated into rational multi-gene attenuation strategies aimed at balancing viral replication and immunogenicity. Further experimental validation would be required to determine the feasibility of such approaches.

## Supplementary Information

**Additional file 1. Screening of ASFV proteins that downregulate MDA5-induced activation of IFN-β promoter. **293T cells were co-transfected with pCAGGS-HA-MDA5 (200 ng), pGL3-Basic-IFN-β-Luc (200 ng), pCMV-RL (2 ng), along with expression plasmids encoding ASFV proteins (400 ng) or empty vector. Luciferase activity was measured 24 hours after transfection.

**Additional file 2. pC147L downregulates the antiviral signaling induced by both HMW and LMW poly(I:C). **PK-15 cells transfected with either pcDNA3-2xFlag-C147L (1 μg) or pcDNA3-2xFlag (1 μg) for 24 hours were subsequently transfected with HMW or LMW poly(I:C) (2 μg/mL) for 2 hours. RT-PCR was performed to assess the transcript levels of immune cytokines, C147L, and β-actin.

## Data Availability

All data generated or analysed during this study are included in this published article.
