# Supplementary figures and images for: African swine fever virus pC147L inhibits retinoic acid-inducible gene I-like receptors pathway via targeting MAVS-TRAF6 complex
Source: Vet Res. 2026 Mar 23;57:58. doi: 10.1186/s13567-026-01716-y (PMC13126741; doi:10.1186/s13567-026-01716-y)

293T

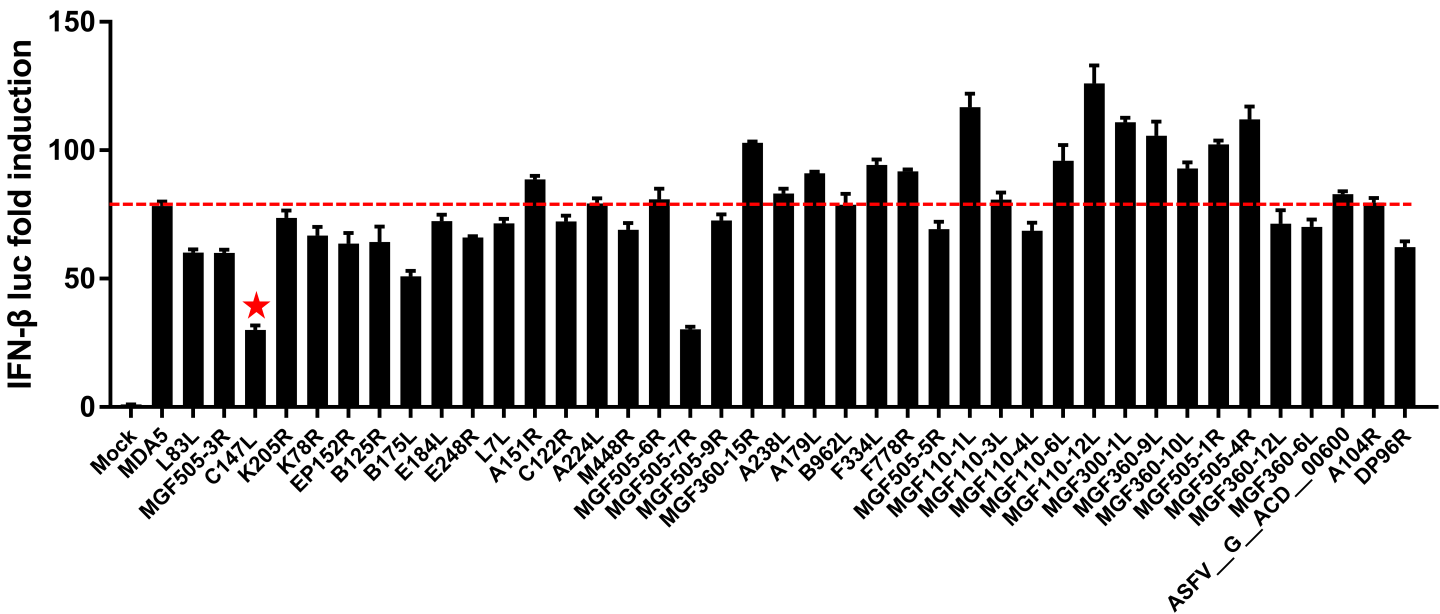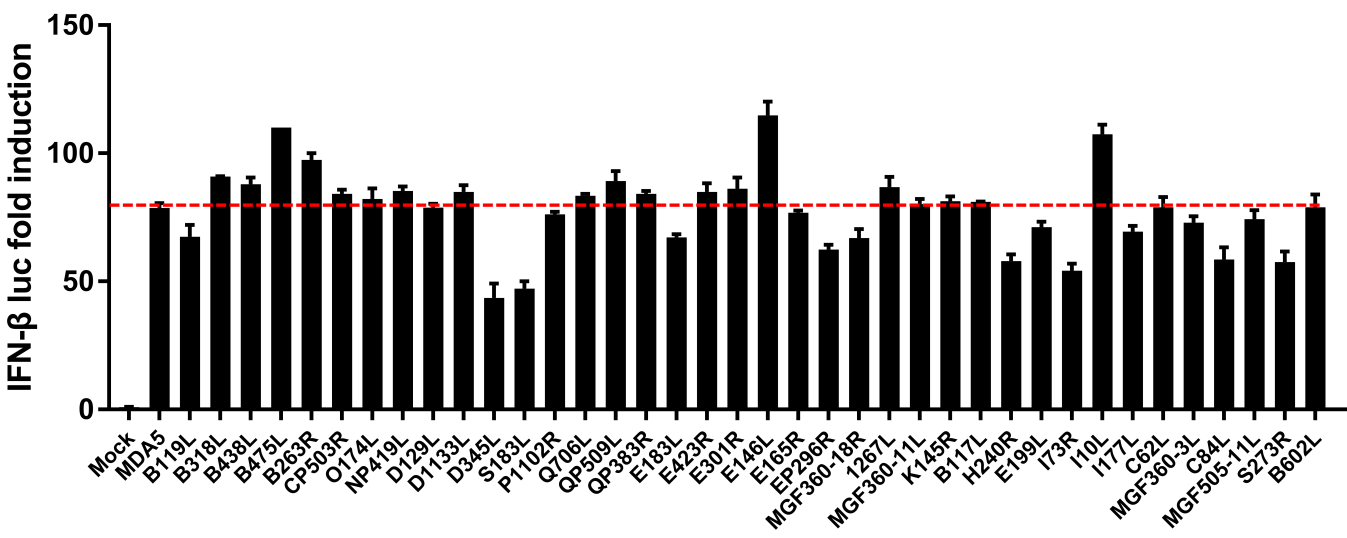

Supplement: Supplementary file 1 — Additional file 1. Screening of ASFV proteins that downregulate MDA5-induced activation of IFN-β promoter. 293T cells were co-transfected with pCAGGS-HA-MDA5 (200 ng), pGL3-Basic-IFN-β-Luc (200 ng), pCMV-RL (2 ng), along with expression plasmids encoding ASFV proteins (400 ng) or empty vector. Luciferase activity was measured 24 hours after transfection. [file 13567_2026_1716_MOESM1_ESM.pdf]

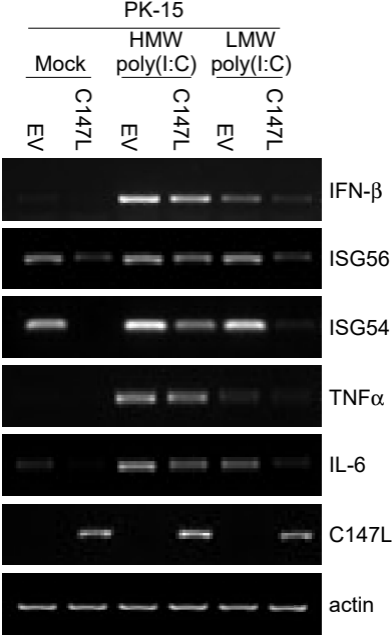

Supplement: Supplementary file 2 — Additional file 2. pC147L downregulates the antiviral signaling induced by both HMW and LMW poly(I:C). PK-15 cells transfected with either pcDNA3-2xFlag-C147L (1 μg) or pcDNA3-2xFlag (1 μg) for 24 hours were subsequently transfected with HMW or LMW poly(I:C) (2 μg/mL) for 2 hours. RT-PCR was performed to assess the transcript levels of immune cytokines, C147L, and β-actin. [file 13567_2026_1716_MOESM2_ESM.pdf]
